# Identification of Prognostic Markers and Potential Therapeutic Targets in Gastric Adenocarcinoma by Machine Learning Based on mRNAsi Index

**DOI:** 10.1155/2022/8926127

**Published:** 2022-09-30

**Authors:** Si Hong Guo, Li Ma, Jie Chen

**Affiliations:** ^1^Personal Health Management, Hong Kong Baptist University, Hong Kong 999077, China; ^2^Department of Gynaecologic Oncology, Harbin Medical University Cancer Hospital, Harbin 150000, China

## Abstract

**Background:**

Cancer stem cells (CSCs), characterized by self-renewal and therapeutic resistance, play important roles in stomach adenocarcinoma (STAD). However, the molecular mechanism of STAD stem cells is still unclear. In this study, our purpose is to explore the expression of stem cell-related genes in STAD.

**Methods:**

The stemness index based on mRNA expression (mRNAsi) was used to analyze STAD cases in The Cancer Genome Atlas (TCGA). Firstly, mRNAsi was used and analyzed by differential expression, survival analysis, clinical stage, and gender in STAD. Then, weighted gene coexpression network analysis (WGCNA) was used to discover the fascinating modules and key genes. Enrichment analysis was carried out to annotate the functions and pathways of key genes. The gene expression comprehensive database (GEO) in STAD was used to verify the expression levels of key genes in all cancers. Protein-protein interaction networks is used to determine the relationships between key genes.

**Results:**

The mRNAsi was obviously upregulated in tumor cases. With the increase of tumor stage and T stage, the mRNAsi score decreased, and the overall survival rate of high score group patients was better. According to the degree of association with mRNAsi, different modules and key genes were screened out. A total of 6,740 differential genes were found, of which 1,147 genes were downregulated and 5,593 genes were upregulated. 19 key genes (BUB1, BUB1B, KIF14, NCAPH, RACGAP, KIF15, CENPF, TPX2, RAD54L, KIF18B, KIF4A, TTK, SGO2, PLK4, ARHGAP11A, XRCC2, Clorf112, NCAPG, and ORC6) were screened due to significant upregulation in STAD. And they had been proven that enriched from the cell cycle Kyoto Encyclopedia of Genes and Genomes (KEGG) pathway, relating to cell proliferation Gene Ontology (GO) terms, as well. Among them, 9 genes have been extensively associated to OS, and 3 genes had been associated to receive chemotherapy resistance. PPI protein network suggests that there is a sturdy correlation between these key genes.

**Conclusion:**

A total of 19 key genes were found to play an essential position in retaining the traits of STAD stem cells. These genes can be used to evaluate the prognosis of STAD patients or become specific therapeutic targets.

## 1. Introduction

The incidence rate and mortality of stomach cancer decreased significantly in five years, but it still ranked third among common malignant tumors and the second leading cause of cancer-related death [1]. Ninety percent of all tumors of the stomach are malignancies, and stomach adenocarcinoma (STAD) accounts for 95% of all cases of malignancies [2].

In current years, the characteristic of most cancers stem telephone has been mentioned such as self-renewal and unlimited proliferation [3–5]. CSC theory points out that tumor proliferation, therapeutic resistance, and recurrence are additionally pushed by way of a small range of tumor stem cells hidden in most cancers. It explains these clinical observations, such as tumor recurrence, tumor dormancy, and metastasis after successful surgical resection, chemotherapy, and radiotherapy [6]. CSCs have been found in several human malignancies, such as leukemia [7], breast cancer [8], colorectal cancer [9], and brain cancer [10]. In addition, strong preclinical data and clinical evidence have been added as supports of the existence of gastric CSCs [11]. Therefore, CSC research is able to provide a new paradigm for managing patients with STAD.

A growing number of studies have shown cancer stemness is associated with being transcriptomic, genomic, epigenomic, and proteomic [12]. Within the last decade, The Cancer Genome Atlas (TCGA) has elucidated the primary tumor landscapes by generating comprehensive multiomics characteristics, along with pathophysiological feature and clinical information annotations [13]. Machine learning has been increasing applied in various areas of society and has become a useful strategy in biotechnology [14]. Tathiane et al. used publicly available molecular profiles from TCGA to obtain two independent stemness indices by using original one-class logistic regression machine-learning algorithm (OCLR) to complete the integration of transcriptome, methylome, and transcription factor [15]. One was mDNAsi which reflects epigenetic features; the other was mRNAsi which reflects gene expression. Malta et al. identified the relationship between the two stem cell indices and new carcinogenesis pathways, somatic cell changes, microRNAs (miRNAs), and transcription regulatory networks. These characteristics are related to cancer stem cells in specific molecular subtypes of TCGA tumors, which may be the factors controlling cancer stem cells. Importantly, higher stem cell index value is related to the active biological processes and greater tumor dedifferentiation in tumor stem cells, as reflected in histopathological grade. Metastatic tumor cells show more dedifferentiation in phenotype, which may contribute to their invasiveness. The stemness indices had positive correlation with tumor dedifferentiation and biological active in CSCs [16]. The mRNAsi and mDNAsi scores in TCGA samples had been calculated by applying the stemness indices.

Weighted gene coexpression network analysis (WGCNA), a method commonly used to explore biological networks, paired relationships between genes and phenotypes. WGCNA transforms gene expression data into coexpression module, providing insights into signaling networks, and mine the pathway-related modules [17]. It is widely applied in many physiological and pathological processes, including cancer, genetic therapy, and clinical data analysis, which can be useful for identifying biomarkers of disease or target points for therapy [18, 19].

In this study, our purpose is to identify key genes associated with STAD stemness in TCGA based on mRNAsi scores. The purpose of this study was to provide an interesting bioinformatics method for identifying stem cell-related genes and revealing the role of some CSC-related genes in STAD.

## 2. Materials and Methods

### 2.1. Software and R Packages

We used R Studio version 1.2.5042 (URL: https://rstudio.com/) with R version 3.6.2 (URL: https://www.r-project.org/) in this study. The programming software Perl version 64-bit (URL: https://www.perl.org/) was used for data processing. All R packages were downloaded from Bioconductor (URL: https://www.bioconductor.org/).

### 2.2. Database and mRNAsi Index

The RNA-sequencing (RNA-seq) of STAD and all pathological and clinical information were downloaded from TCGA database (URL: https://portal.gdc.cancer.gov/). These data were updated on 5 October 2019. The results of RNA-seq were including 375 cancer samples and 32 normal samples, structured for a matrix file. We used Ensemble data to exchange the gene names expressed by Ensembl IDs which are specifically converted into a gene symbol matrix. Moreover, to explore the mode of action of CSC-related genes in chemotherapy resistance, we download the microarray (GSE14210) results from the Gene Expression from the Gene Expression Omnibus (GEO) (URL: https://www.ncbi.nlm.nih.gov/geo/). We referred to the mRNAsi index data for all types of tissues in the supporting documents to Malta et al.'s article and specifically screened the mRNAsi index of patients with stomach adenocarcinoma for incorporation into TCGA data for stomach adenocarcinoma, with the unmatched cases deleted.

### 2.3. 2.3 Differential Expressed Gene (DEG) Analysis

We used the R package “limma” for differential expression analysis [20]. We used the cut-off values, which were fold change > 1 and adj.*P* < 0.05, to screen for DEGs between normal health and stomach adenocarcinoma samples. The volcano plot and the box-plots showing differences in key genes presented in this study were drawn by the R package “pheatmap” and “ggpubr,” respectively.

### 2.4. WGCNA

WGCNA was performed using the WGCNA R package [17], which were “matrixStats,” “Hmisc,” “foreach,” “doParallel,” “fastcluster,” dynamicTreeCut,” “survival,” and “WGCNA.” Before the building of coexpression network, the rectangular Euclidean relative distance of every take a look at pattern was once calculated by means of practical adjacency method, and the integration connectivity of the total pattern community calculated via distance was once standardized via practical scaling method. Due to some exceptional genes with no tremendous trade in expression between samples which are surprisingly correlated in WGCNA as a whole, it appears that the genes with the most biased expression have been used in the subsequent WGCNA analysis. The gene with the highest DEG variance of 25% was selected. Clear ordinary value pattern information with connectivity is much less than -2.5. Function pickSoftThreshold was used to calculate scale-free topology becoming indices*R*^2^corresponding to one-of-a-kind smooth thresholding powers*β*. The*β*value was used as lengthy as*R*^2^reaching 0.8. After that, the gene expression matrix was converted into an adjacency matrix and a Topological Overlap Matrix (TOM), and then the corresponding dissimilarity of TOM (dissTOM) was calculated. For module detection, hierarchical clustering was used to produce a hierarchical clustering tree (dendrogram) of genes by using characteristic “hclust” based totally on dissTOM. The Dynamic Tree Cut approach was carried out for department reduction to generate modules. During this, a quite massive minimal module measurement of minClusterSize = 30 to department splitting had been chosen to avoid producing too many small or massive modules. To consider the magnitude of every module, gene significance (GS) was once calculated to measure the correlation coefficient between genes and pattern traits. The module eigengene (ME) is described as the first foremost thing of a given element and can be regarded as a consultant of the gene expression profile of the module integration. It was calculated by using purposeful module genes. If their MES correlation coefficient is higher than 0.75, the modules will be merged, with capacity that they have considerable comparable gene expression levels. Here, we can pick out mRNAsi and epigenetically regulated mRNAsi as scientific phenotypes.

After selecting the components of interest, let us calculate the GS and module membership of each key gene (MM, the significance between the module's own gene and gene expression profile), and set their threshold values. The thresholds for screening key genes in the module were defined as cor.gene MM > 0.8 and cor.gene GS > 0.5.

### 2.5. Overall Survival Curve

Finally, to determine the prognostic significance and value of mRNAsi scores, we can draw the Kaplan–Meier diagram of mRNAsi index to explore the overall survival deviation between patients with low and high mRNAsi index. In this part, R package “survival” and “surviminer” were used, and a log-rank test is used to test the relationship between them. In key gene validation analysis, Kaplan–Meier survival curves of key genes were drawn with the online tool Kaplan–Meier plotter [21] (URL:http://www.kmplot.com/analysis/index.php?p=service).

### 2.6. Functional Annotation Gene Ontology (GO) and Kyoto Encyclopedia of Genes and Genomes (KEGG) Analyses

The GO functional annotations and KEGG pathway enrichment analysis shown in this study were obtained from the data analysis conducted by the R package “cluster Profiler” to investigate the biological functions of key genes. The threshold values were as follows: *P* < 0.01 and FDR < 0.05.

### 2.7. Gene Coexpression Analysis and Construction of Protein-Protein Interaction (PPI) Network

In order to further study the stability of these special relationships at the transcriptional level, we calculated the coexpression relationships among key genes within the module depending on the gene expression level. The R “corrlot” package is mainly used to calculate the Pearson correlation degree between genes. The STAD data set was selected from TCGA for analysis and research, and the routine data were analyzed by the Pearson correlation test. Results with a correlation coefficient > 0.3 and *P* value < 0.01 were considered statistically significant.

Accurately retrieve PPI network from STRING version 11.0 (URL: https://string-db.org/) [22]. And display the bar graph of the nodes in the network with top-level network connectivity. The minimum required interaction score was set to a medium confidence of 0.4, and now, the hidden branch nodes in the network are disconnected. The number of adjacent nodes of each gene in the PPI network was calculated, and then, the genes were sorted by the bar graph combined with the number of adjacent nodes.

## 3. Results

### 3.1. Clinical Characteristics of mRNAsi and DEGs in STAD

**Figure 1:**
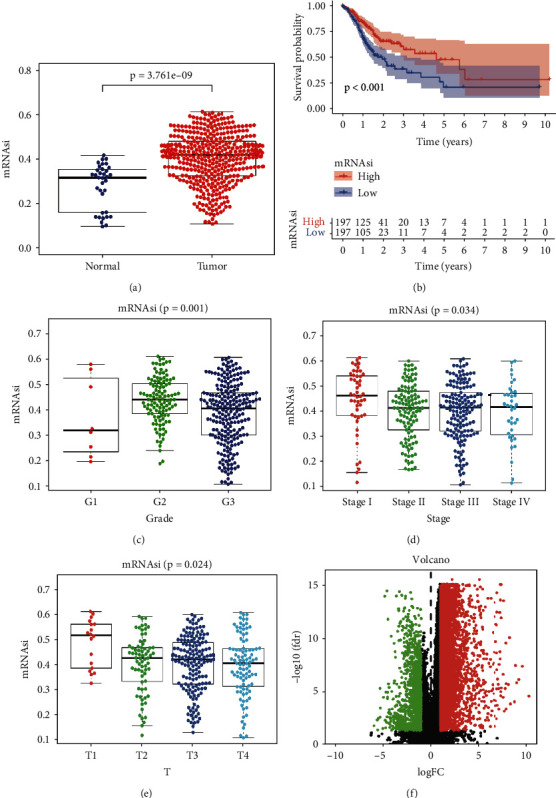
The correlation of mRNAsi profiles with STAD. (a) Scatter plot illustrating the difference of mRNAsi index expression between normal tissues and tumors. (b) Kaplan–Meier survival curve of correlation between mRNAsi score and OS of STAD patients. Detect the correlation between mRNAsi score and the Grade (c), Stage (d), and T degree (e) by the Kruskal-Wallis test. (f) Volcano map of DEGs between STAD tissues and normal tissues. Downregulated genes are indicated in green, and upregulated genes are indicated in red. STAD: stomach adenocarcinoma; DEGs: differentially expressed genes.

The mRNAsi is an index of CSCs that can quantitatively describe the similarity between tumor cells and stem cells. We observe large distinction in mRNAsi between tumor and ordinary tissues (Figure 1(a)). In the survival analysis, we divided gastric cancer patient into higher mRNAsi score group and lower mRNAsi group by using mRNAsi median value. Obviously, patients with higher mRNAsi scores have greater overall survival in contrast with sufferers with lower mRNAsi scores (Figure 1(b)), the five-year survival rate of higher scores group is 47.9% with CI (0.344, 0.668), and the lower scores group is 21.2% with CI (0.107, 0.421). Surprisingly, the mRNAsi scores tend to decline with the grade increasing with the exception of G1 (only 8 samples). Also, the mRNAsi score shows an overall decreasing trend in stage and T (Figures 1(c), 1(d), and 1(e)). The Kruskal Wallis test was once used to determine the value of variations between groups.

We download mRNA-seq data and did difference analysis to compare STAD and normal since the mRNAsi difference between tumor and normal. We find 6740 DEGs in which 1147 were downregulated and 5593 were upregulated (Figure 1(f)).

### 3.2. WGCNA: Identifying the Most Significant Modules and Genes

**Figure 2:**
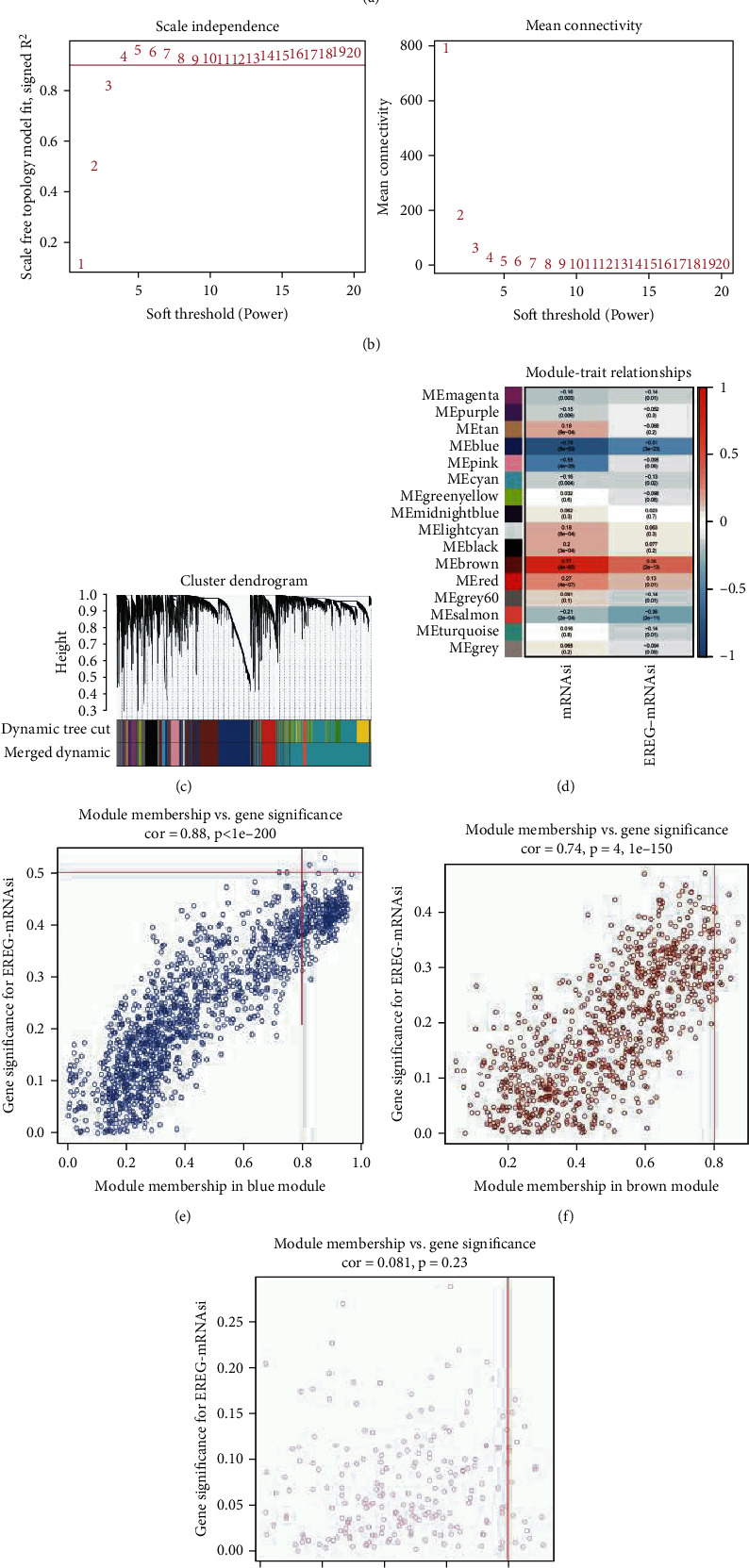
Construction of weighted gene coexpression network for STAD stemness related datasets. (a) Identify and remove outlier samples through average linkage hierarchical clustering. Samples exceeding the red line were considered deviations in gene expression. (b) Network topology analysis of different soft threshold powers. The left figure shows the influence of soft threshold power on the scale-free topological fitting index. The right figure shows the influence of soft threshold power on average connectivity. (c) Clustering dendrograms was done via mean linkage hierarchical. (d) Module-trait relationships. Each column represents a clinical phenotype, and each row denotes an ME. The correlation coefficient and *P* value are contained in each cell. (e–g) Scatterplots of GS for weight vs. MM to pick out the key genes from the blue, brown, and pink modules. STAD: stomach adenocarcinoma; ME: module eigengene; GS: gene significance; MM: module membership.

With WGCNA, we built a gene coexpression network to become aware of biologically significant gene modules. It can help us to understand the genes associated with STAD stemness. We put 6740 DEGs with the highest variance of 25% into the same module through cluster analysis. Before that, the outlier samples should be removed (Figure 2(a)). According to the lowest value of scale-free network, the *β* value is determined. What the pickSoftThreshold function does is to find the appropriate power. The selection of the power value is determined by *β* value. Calculate the correlation intensity (weighted correlation value) of expression levels among all genes to obtain the adjacency matrix. As a result, we choose *β* = 4 (scale-free *R*^2^ = 0.9) as the soft threshold (Figure 2(b)). We find 16 modules for subsequent evaluation (Figure 2(c)).

Taking MS as the total gene expression level of the corresponding module, the correlation between MS and clinical phenotype was calculated. This is useful for us to discover the relationship between these modules and the dryness index of the sample. By calculating the Pearson correlation coefficient, a threshold value can be obtained. If the correlation coefficient is greater than 0.8 or so, it can be used as the basis of strong correlation between the two genes. The consequences confirmed that the blue and brown modules were extensively correlated with mRNasi, and the correlation was once close to 0.8. However, the correlation coefficient of the brown module is 0.77, which is higher. In addition, the pink module was fantastically negatively correlated with mRNasi (Figure 2(d)). Therefore, the brown module was chosen through us as the most fascinating module for subsequent analysis.

The threshold for screening key genes in the mRNAsi group was described as cor.MM > 0.8 and cor.GS > 0.5. We pick 19 key genes (BUB1, BUB1B, KIF14, NCAPH, RACGAP1, KIF15, CENPF, TPX2, RAD54L, KIF18B, KIF4A, TTK, SGO2, PLK4, ARHGAP11A, XRCC2, Clorf112, NCAPG, and ORC6), as shown in Figures 2(e)–2(g). And we exhibit the distinct expressions of key genes between most cancers and ordinary samples in TCGA; all these genes in brown module are upregulated in tumor cases (Figure 2(f)).

### 3.3. Enrichment Analysis of Brown Module

**Figure 3:**
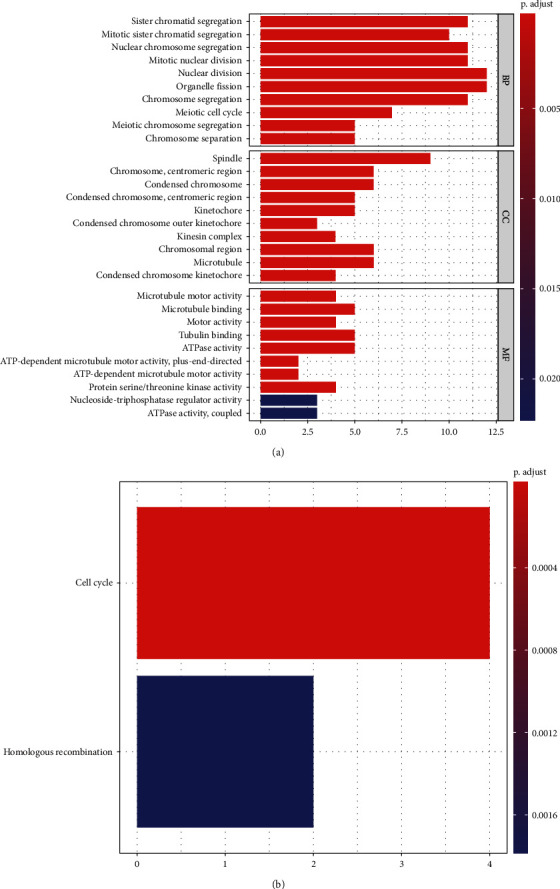
Functional analysis of brown module. (a) GO enrichment analysis. (b) KEGG enrichment analysis. GO: Gene Ontology; KEGG: Kyoto Encyclopedia of Genes and Genomes; BP: biological process; CC: cellular component; MF: molecular function.

We use GO and KEGG analysis to elucidate the function similarities of module brown genes. The results show that nuclear division, spindle, and microtubule binding are the most great enrichments in cellular component (CC), biological process (BP), and molecular function (MF) groups (Figure 3(a)). KEGG pathway enrichment analysis suggested cell cycle and homologous recombination pathways are significant pathways (Figure 3(b)). All of them are related to cancer stem cells.

### 3.4. Data Validation

**Figure 4:**
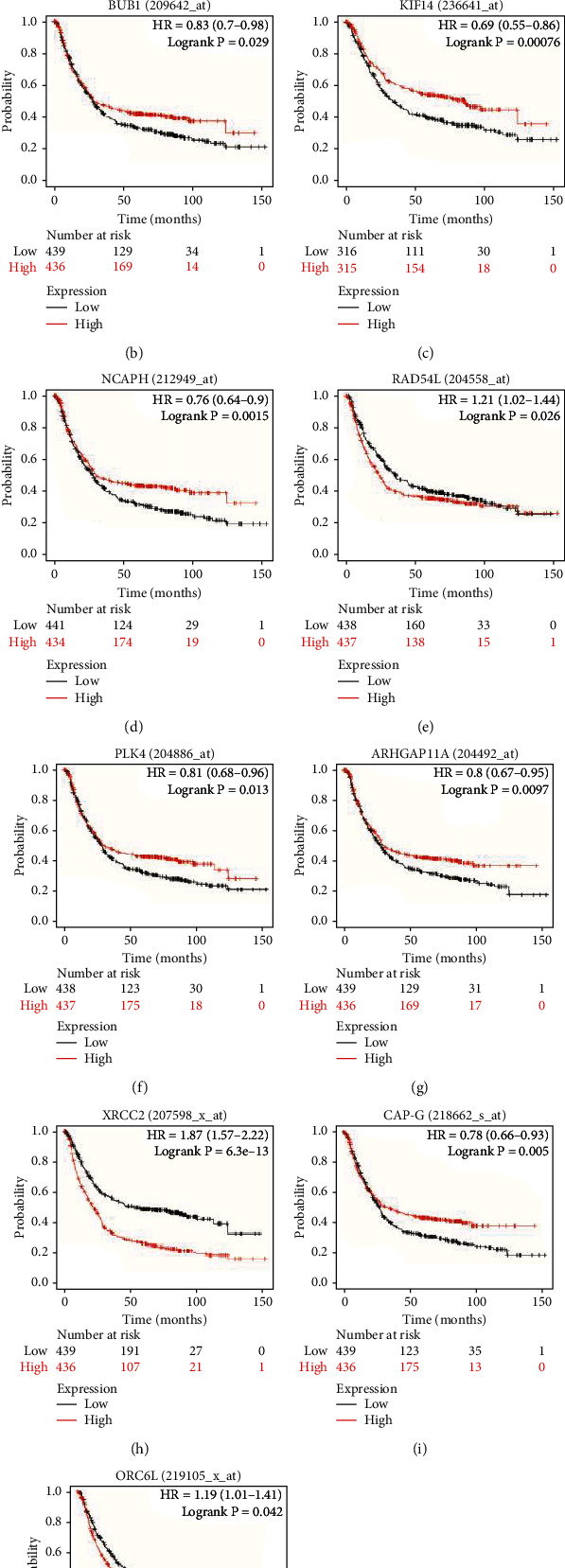
Verification of the influence of key genes on diseases. (a) The mRNA expression level of key genes between tumor and normal tissues in TCGA STAD dataset. The Kaplan–Meier plotter database was used to assess the correlation between the expression of BUB1 (b), KIF14 (c), NCAPH (d), RAD54L (e), PLK4 (f), ARHGAP11A (g), XRCC2 (h), NCAPG (i, also called CAP-G), ORC6 (j, also called ORC6L), and the OS of STAD patients. Kaplan–Meier survival plots (K–M plots) were generated using the on-line tool, Kaplan–Meier plotter. (k) Venn diagram of the relationship between 19 key genes and acquired chemoresistance by GSE14210.

Firstly, the STAD dataset of TCGA showed significant differences in the expression of all key genes between normal and tumor cases (Figure 4(a)). In all patients with STAD, the Kaplan–Meier curve and log rank test analysis showed that 7 genes in the brown module were significantly associated with OS (*P* < 0.05, FDR < 0.05) (Figures 4(b)–4(j)).

It is well known that CSCs have chemoresistance, and resistance is related to cancer-associated fibroblasts in the extracellular matrix [23]. The mapping of GSE14210 is based on Venn diagram. 19 key gene maps selected from the brown module were scored by GS and MM scores. Finally, SGO2, TTK, and CENPF were associated with the acquired chemoresistance to cisplatin and fluorouracil combination chemotherapy in gastric cancer (Figure 4(k)).

### 3.5. Protein-Protein Interactions (PPI) among Genes of Brown Module

**Figure 5:**
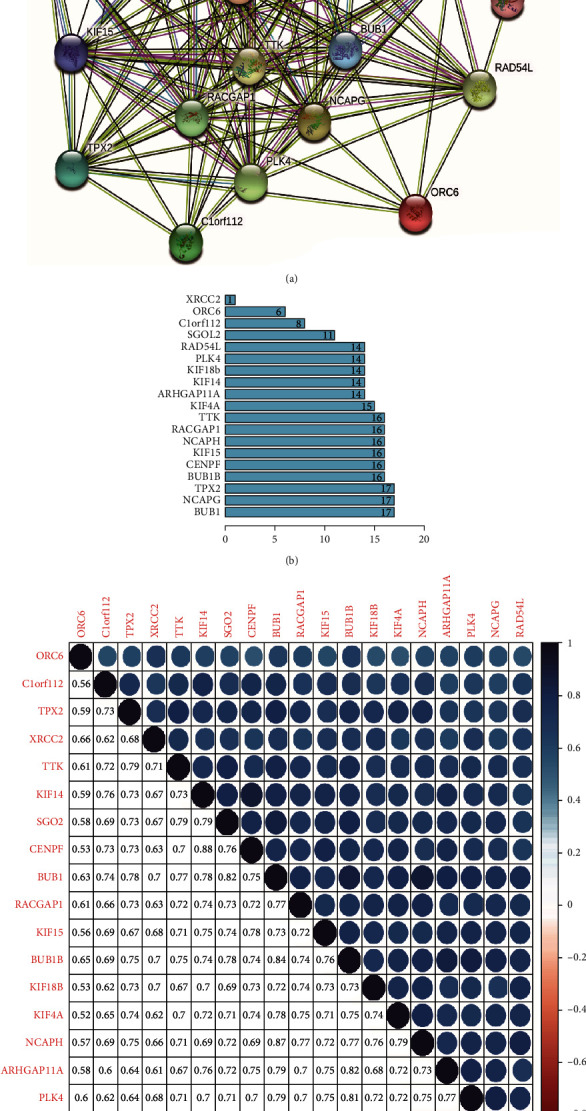
PPI interactive network. (a) String diagram composed of 19 key genes as nodes. (b) The bar-plot lists the connections of key genes in the brown module by the counts of connections. (c) Correlation analysis between key genes. The higher phase of the graph indicates the degree of correlation. The darker the color, the greater the correlation. The lower part shows the corresponding correlation value. PPI: protein-protein interaction.

The application of the on-line device STRING (URL: http://string-db.org/) to protein-protein interaction networks for each module will assist us to explore the interplay between key genes extra deeply. There were 19 nodes and 129 edges in the shaped PPI network, and the PPI enrichment (*P* value < 0.01) (Figure 5(a)). In addition, the significant nodes shown in the bar-plot can identify the genes most closely related to other members of the module (Figure 5(b)).

The correlation between the key genes of this module was strong (Figure 5(c)), and the correlation was statistically significant (*P* < 0.01). The correlation between CENPF and ORC6 (0.53), KIF18B, and ORC6 (0.53) was the lowest, whereas the correlation between CENPF and KIF14 (0.88) was the highest.

## 4. Discussion

The morbidity and mortality of gastric most cancers stay excessive all over the worldwide. In current years, CSCs have been mentioned to make vital contributions to tumor progression, recurrence, and therapeutic resistance [24, 25]. Therefore, therapy concentrating on STAD stem cells is essential. In addition, choosing out the emergence of these druggable genetic ameliorations in pancancer cases, and whether or not there are modifications in the expression of the equal mRNAsi-related genes, is additionally a query priceless of dialogue in the future work.

In this study, we tried to discover key genes associated to STAD stem cells in TCGA database. We used WGCNA based totally on mRNAsi scores, as calculated by Salomonis et al. [26]. The tumor case had a greater mRNAsi rating than the regular case. The mRNAsi scores reduced with the sickness grade, stage, and T stage, though the mRNAsi rating of G1 was once small which may also be associated to inadequate pattern size. The excessive mRNAsi team confirmed a decrease survival chance than the low team in the first 5 years, which used to be constant with the negative consequence related with stemness features.

We developed coexpression modules through WGCNA and pick out brown module as the best correlations with mRNAsi. Key genes have been screened from the blue module primarily based on the GS and MM scores. The expression degrees of key genes are appreciably upregulated in tumor samples. There have been robust coexpression relationships at the transcriptional degree in brown module. There was additionally a robust PPI community among proteins of this module. The key genes intently associated to pluripotent stem cells have been confirmed to be overexpressed in most tumors. Moreover, all organ tissues are developed from pluripotent stem cells, suggesting that key genes might also play a position in keeping stem cellphone residences in a range of cancers. The consequences led us to reassess the relationship between CSC traits and STAD progression.

Undifferentiated major tumors are more probably to reason most cancer cells to unfold to far-off organs, mainly to sickness development and negative prognosis. Moreover, CSCs are typically resistant to handy remedies [27]. The acquisition of progenitor cell-like and stem cell-like traits and loss of the differentiated phenotype are manifestations of most cancer development [28], regular with the expand in STAD stemness as the tumor progression. In our study, we observed that sufferers with greater corrected mRNAsi rankings had decreased ordinary survival rates, which used to be regular with the negative prognosis related with CSC characteristics. Disease stage 1 and T1 stage STAD had pretty greater CSC characteristics, indicating the stem telephone residences start to upward thrust from initiation of the cancer.

Functional annotations of the brown module had been chiefly associated to the stem cell self-renewal and proliferation characteristics. The pathway enrichment advised that the four key genes in the cycle pathway time period have been most possibly a useful gene set that impacts tumor stemness via regulating the cell cycle.

The gene units that keep the traits of stem cells in a range of cancers may additionally have similarities. Since the formation of a range of organ tissues takes place from pluripotent stem cells, their CSCs are dedifferentiated with stem mobile phone characteristics. This reverse improvement has made a range of CSCs possessing some traits of pluripotent stem cells. Moreover, their stage of overexpression was once associated to the stage of stemness, and their persisted expand might also promote modifications in tumor development and posttherapy progression. More than half of the key genes have been mentioned in STAD, and some have been proven to be related with the traits of CSCs. BUB1 is related with the most cancer stem cell attainable in breast cancer [29]. An issue highlights a study that links the presentation of kinetochores within mitosis to an essential requirement for BUB1 threonine kinase B (BUB 1B), broadening our understanding of the cell-cycle machinery in CSCs [30]. Kinesin family member 15 (KIF15) promotes the CSC phenotype and malignancy by means of PHGDH-mediated ROS imbalance in hepatocellular carcinoma [31]. TTK gene was overexpressed in the CSC-like cell populace remoted from human esophageal carcinoma phone strains as properly as in the human more than one myeloma stem cells sorted through aldehyde dehydrogenase 1 (ALDH1) [32, 33].

Survival curves have been generated to validate the prognostic fee of the key genes in brown module in STAD. In the K-M plots, 7 genes had been substantially related with prognoses (*P* < 0.01, FDR < 0.05). High expression of BUB1, TPX2, and X-ray repair cross complementing 2 (XRCC2) had been noticeably related with negative prognoses. The expression of NCAPH, NCAPG, RACGAP1, and SGO2 has been positively correlated with affected person prognosis. As known, CSCs can withstand clinical remedy and make contributions to tumor relapse. The key genes had been validated in GSE14210, and SGO2, TTK, and CENPF have been related with the obtained chemoresistance to cisplatin and fluorouracil mixture chemotherapy in gastric cancer. Several studies had proven that CSCs have one or greater abnormalities in signaling pathways that modify self-renewal. The Wnt/*β*-catenin, Notch, and Hedgehog pathways have been mentioned fully [34]. Wnt/*β*-catenin KIF14, TPX2, KIF18B, and PLK4 in the Wnt/*β*-catenin pathway [35–38], TTK and XRCC2 in the Hedgehog pathway [37, 38], and RACGAP1 and TTK in the Notch pathway [39, 40] may additionally be necessary for the tumorigenicity of CSCs. These genes are vital therapeutic aimed at inhibiting the self-renewal, proliferation, and tumor development of CSCs.

## 5. Conclusions

In summary, 19 key genes have been determined to play necessary roles in STAD stem phone maintenance. The validations confirmed that these genes ought to be beneficial for outlining the prognosis of STAD patients. These genes may also be therapeutic pursuits for inhibiting STAD stemness characteristics. However, our conclusions are primarily based on the retrospective information, and similarly organic and scientific investigation of these genes should lead to novel insights into the manageable associations of CSCs with a STAD prognosis.

## Data Availability

The datasets generated and/or analyzed during this research period can be obtained from the corresponding author.
